# Supplementary material for: HIVprotI: an integrated web based platform for prediction and design of HIV proteins inhibitors
Source: J Cheminform. 2018 Mar 9;10:12. doi: 10.1186/s13321-018-0266-y (PMC5845081; doi:10.1186/s13321-018-0266-y)
Supplement: Supplementary file 2 — Additional file 2. Source code of HIVProtI web server. [file 13321_2018_266_MOESM2_ESM.zip › HIVprotI_Source-code/hivprotI/weka-3-6-6/doc/weka/core/CheckScheme.PostProcessor.html]

CheckScheme.PostProcessor


---


|  |  |  |
| --- | --- | --- |
| **Overview** \| **Package** \| **Class** \| **Tree** \| **Deprecated** \| **Index** \| **Help** \| **Weka's home** | |  |
| **PREV CLASS**   **NEXT CLASS** | **FRAMES**    **NO FRAMES**     **All Classes** |  |
| SUMMARY: NESTED \| FIELD \| CONSTR \| METHOD | DETAIL: FIELD \| CONSTR \| METHOD |  |


---


## weka.core Class CheckScheme.PostProcessor

```
java.lang.Object
  weka.core.CheckScheme.PostProcessor
```

**All Implemented Interfaces:**: RevisionHandler

**Enclosing class:**: CheckScheme

---

``` public static class CheckScheme.PostProcessor extends java.lang.Object implements RevisionHandler ```

a class for postprocessing the test-data

---

**Constructor Summary**

`CheckScheme.PostProcessor()`


| **Method Summary** | |
| --- | --- |
| `java.lang.String` | `getRevision()`             Returns the revision string. |
| `Instances` | `process(Instances data)`             Provides a hook for derived classes to further modify the data. |

**Methods inherited from class java.lang.Object**

`equals, getClass, hashCode, notify, notifyAll, toString, wait, wait, wait`

**Constructor Detail**

### CheckScheme.PostProcessor

```
public CheckScheme.PostProcessor()
```


**Method Detail**

### process

```
public Instances process(Instances data)
```

:   Provides a hook for derived classes to further modify the data. Currently,
    the data is just passed through.

    :   **Parameters:**: `data` - the data to process **Returns:**: the processed data

---


### getRevision

```
public java.lang.String getRevision()
```

:   Returns the revision string.

    :   **Specified by:**: `getRevision` in interface `RevisionHandler`
    :   **Returns:**: the revision


---


|  |  |  |
| --- | --- | --- |
| **Overview** \| **Package** \| **Class** \| **Tree** \| **Deprecated** \| **Index** \| **Help** \| **Weka's home** | |  |
| **PREV CLASS**   **NEXT CLASS** | **FRAMES**    **NO FRAMES**     **All Classes** |  |
| SUMMARY: NESTED \| FIELD \| CONSTR \| METHOD | DETAIL: FIELD \| CONSTR \| METHOD |  |


---
